# From Antigen Delivery System to Adjuvanticy: The Board Application of Nanoparticles in Vaccinology

**DOI:** 10.3390/vaccines3040930

**Published:** 2015-11-05

**Authors:** Diana Boraschi, Paola Italiani

**Affiliations:** 1 Institute of Protein Biochemistry, National Research Council, Via Pietro Castellino 111, I-80131 Napoli, Italy; E-Mail: p.italiani@ibp.cnr.it; 2 Institute of Biomedical Technologies, National Research Council, Via Fratelli Cervi 93, 20090 Segrate (Milano), Italy

**Keywords:** nanoparticles, vaccines, adjuvants, inflammation

## Abstract

In the last years, nanotechnologies have raised great interest because of the potential applications of engineered nanoparticles in nanomedicine (*i.e.*, in vaccination, in diagnostic imaging procedures, and as therapeutic drug delivery systems). The use of nanoparticles in medicine has brought about the issue of their interaction with the immune system for two main reasons: first, understanding how long nanomedicines could persist in the organism and exert their beneficial effects before being recognized and eliminated by our defensive systems; second, understanding how the immune responses can be modulated by nanoparticles in order to obtain optimal effects. This issue is crucial in vaccine formulations based on the use of nanoparticles, which can operate both as a delivery system to enhance antigen processing and as an immunostimulatory adjuvant to induce and amplify protective immunity, in part because of their ability to activate the inflammasome and induce the maturation of interleukin 1β. Nanoparticles can be excellent adjuvants due to their biocompatibility and their physicochemical properties (e.g., size, shape, and surface charge), which can be tailored to obtain different immunological effects. This review provides an overview of recent strategies for the use of nanoparticles as promising/attractive adjuvants for novel prophylactic and therapeutic vaccines. The use of nanovaccines, with their practically infinite possibilities of specific design, could open the way to precision vaccinology, *i.e.*, vaccine formulations tailored on the individual immune reactivity status.

## 1. Introduction

The use of nanoparticles (NPs) in medicine has a very important and promising application in vaccinology, both as an antigen carrier and as an adjuvant [1]. Since NP-based vaccines need immune recognition, their design is tailored in order to improve their uptake by immune cells and their ability to target specific intracellular compartments, which can be achieved by modification of material, shape, size, and surface charge. Particulate vaccine formulations have been used for decades, with bread crumbs, to kill bacterial particles, and oil emulsions are being used to increase antigen persistence and amplify the immune response (reviewed in [2]). It is noticeable that alum, which has been used in human vaccines since the 1920s, is a particulated preparation of aluminum salts that attains the double goal of maintaining the antigen in the body for prolonged periods of time (depot effect) and of inducing a localized mild inflammation that initiates a potent protective immune response (adjuvant effect, [2]). Alum was the only allowed adjuvant in human vaccines until the more recent introduction of other types of adjuvanted vaccines, such as the Novartis seasonal influenza vaccine FLUAD^®^ in an oil-in-water emulsion in 1997 [3], and the liposome-based hepatitis A vaccine Epaxal^®^ of Berna Biotec in 2003 [4]. The oil-in-water emulsion MF59 (squalene, Tween 80, sorbitan trioleate; Novartis), after its inclusion in the anti-seasonal influenza vaccine FLUAD^®^, is also included in the vaccines FOCETRIA^®^ (pandemic influenza), AFLUNOV^®^ (pre-pandemic influenza), and OPTAFLU^®^ (influenza A/California/7/2009 (H1N1)pdm09-like strain, A/Texas/50/2012 (H3N2)-derived strain, and B/Massachusetts/2/2012 strain). In 2009, another oil-in-water emulsion, AS03 (Squalene, Tween 80, α-tocopherol; GlaxoSmithKline), was included in the vaccines Pandemrix™ (pandemic influenza) and Prepandrix™ (pre-pandemic influenza). Another particulate adjuvant used in human vaccines since 2005 is AS04, a preparation of the TLR4 agonist monophosphoryl lipid A (MPL) adsorbed on alum particles, which is included in the GlaxoSmithKline vaccines Fendrix^®^ (hepatitis B) and Cervarix^®^ (human papilloma virus) (reviewed in [5]). Several other NP-based adjuvants are in development, such as ADVAX™, composed by semi-crystalline particles of the polysaccharide delta inulin, which is being tested in vaccines against influenza, hepatitis B, and HIV [6,7,8].

Given that the only adjuvanted vaccines for human use rely on particles for improving their efficacy, it is clear why in recent years NP-based vaccines have attracted interest. In this context, a wide range of particles of different chemical composition have been built and used for antigen delivery and as adjuvants in experimental systems. The goal is that of exploiting the possibility of engineering NPs to provide them with a series of desirable characteristics (satisfactory pharmacokinetics, specific cellular targeting, subcellular localization, *etc.*). This will allow us both to improve antigen stability, processing, and immunogenicity, and to modulate the targeting of antigen and molecules to the correct cellular compartments, thereby achieving optimal protective responses and eliminating side effects. Here we will briefly review the state-of-the-art of the use and potential developments of NP-based vaccination strategies.

## 2. Nanoparticles in Antigen Delivery

The major advantage of using particulated antigens in vaccine formulations stems from the fact that efficient antigen presentation and induction of protective immunity require antigen uptake by specialized antigen-presenting cells (APCs). APCs take up foreign materials by endocytosis, process the material in the phagolysosomes, and present the fragments on their surface, bound to major histocompatibility complex (MHC) molecules. This process induces the activation of T lymphocytes and the initiation of adaptive immunity with the generation of immunological memory, which is the basis of protective immunity in vaccination [9]. It is well known that soluble antigens are poorly effective in inducing a good protective immunity, mainly because of the insufficient antigen uptake by APCs. For this reason, soluble vaccine antigens are made more immunogenic by conjugating them to bulkier carriers, a strategy facilitating recognition and uptake by APCs. Conjugation to carriers is a well-developed and effective strategy for inducing/increasing the immunogenicity of poorly immunogenic antigens, such as polysaccharides, for instance, in the case of pneumococcal vaccines [10].

The induction of protective immunity thus implies that the antigen is taken up by APCs, which then can present it to T cells. The two major types of APCs, dendritic cells (DCs) and macrophages, have different functions in the context of the induction of a specific immunity. Both cell types are present in the tissues and can come in contact with foreign molecules/agents, but after antigen uptake only DCs are able to migrate to the lymph nodes and prime naïve T lymphocytes. This implies that DCs are the major APCs involved in the initiation of adaptive and protective immunity, and it also implies that vaccination strategies should specifically target them. On the other hand, tissue-resident macrophages stay in the tissue after antigen uptake, and they are the cells that present the antigen to primed T lymphocytes coming into the tissue in a secondary response [11,12]. Thus, the two types of APCs have different roles in antigen presentation, one being responsible for the induction of protective immunity and memory (the best target of preventive vaccines) and the other responsible for the effective protection of the tissue upon a secondary challenge (*i.e.*, the best target for booster vaccines). The use of nanocarriers able to specifically target one or the other of the two APC types would be of great help in the formulation of vaccines with the desired effects of priming *vs.* challenging protective immunity.

Nanodelivery of vaccine antigens would thus require particles that are readily recognized and taken up by APCs at the site of the inoculum (strategies for hiding them from innate recognition, such as in the stealth particles designed for drug delivery, would be detrimental), biocompatible to avoid damaging or killing the APCs, and able to release the antigen once inside the APCs.

Liposomes, lipid-based vesicles that can be artificially built or can come from cells and microorganisms (exosomes, bacterial particles), are an excellent platform for antigen delivery that can be manipulated to obtain the desired characteristics [13]. Liposomes can carry the microoganisms’ antigens (as in the case of virosomes and bacterial particles) or can incorporate the cargo and act effectively in taking the antigen to the APC, as in the case of autophagosomes and exosomes [14,15,16] and with artificial liposomes [13,17]. The different characteristics of the nanocarriers may allow us to direct them to the correct intracellular compartment so that it can interact with the class I or class II MHC molecules for selective antigen presentation. Because of their flexibility in manipulating their characteristics, their rapid degradation/lack of toxicity, and efficient uptake by APCs with good induction of immunity, liposomes have been studied extensively in the last decades (recently reviewed in [17]). However, although used quite extensively in veterinary vaccinations [17], over 10 years after its approval, Epaxal^®^ is still the only liposome-based vaccine for human use. It is possible that the unsatisfactory stability of many liposomal vaccine formulations might hamper industrial exploitation. Numerous other types of biocompatible particles are being studied for designing optimal antigen delivery, including all those that are used for drug delivery, such as polymeric NPs (such as poly(lactide-co-glycolide-PLGA-particles; [18]) and solid lipid NPs [19,20], proteosomes or outer membrane vesicles [21,22], bacterial spores [23], and bacteriophages [24,25]. None of them, however, has reached the point to be included in vaccine formulations for human use [26,27,28].

However, some vaccines are on the market that use as antigen virus-like particles (VLPs) displaying the major immunogenic antigens of the infectious viruses. These vaccines have revisited the old concept of using killed microorganisms as immunizing agents, and can by all criteria be considered nanoparticle-based vaccines. These include two anti-hepatitis B vaccines (Engerix^®^-B by GlaxoSmithKline and RECOMBIVAX HB^®^ by Merck and Co., Inc.) and two against papillomavirus (GlaxoSmithKline’s Cervarix^®^ and Merck’s GARDASIL^®^).

An important goal when designing antigen delivery in vaccination is the possibility of obtaining cross-presentation, *i.e.*, presentation of an antigen in the context of both MHC-I (typical of viruses and for antigens that enter the cytoplasm) and MHC-II (typical of antigens taken up in phagolysosomes). The two types of presentation induce different types of immune responses, with MHC-I presentation leading mostly to the activation of cell-mediated immunity and MHC-II presentation optimally triggering an antibody response. Since, for most vaccines, both types of responses are desirable, an effective antigen delivery system should promote cross-presentation, *i.e.*, the presentation of the vaccine antigen through both routes [29]. Among nanocarriers that facilitate cross-presentation, fusogenic virosomes have the capacity of fusing with the endosome membrane, thereby releasing their content in the APC cytoplasm [30]. In this way, the antigen carried by virosomes can be presented both in MHC-II (following its endosomal uptake) and in MHC-I (once released in the cytoplasm). Another example, based on a different concept, is represented by a nanoparticulated polysorbitol transporter (PST), containing cationic polyethylenimine (PEI). The antigen-containing particles are taken up in phagosomes and, due to the proton sponge effect of PEI, phagosome swelling and rupture follows with antigen release in the cytoplasm and consequent overall activation of protective immunity [31]. Other types of NPs are designed to preferentially target APCs because of their carbohydrate coat and multivalent glycoconjugate structures. One such particle is the gold glyconanoparticle (GNP), which can establish multiple interactions with receptors on APCs through its sugar coat and which can be loaded with pathogen-specific peptides. Peptide-loaded GNPs can cross-present the antigen and induce both MHC-I- and MHC-II-dependent responses and they were found to effectively immunize against pathogens such as HIV-1, *Listeria monocytogenes*, and *Streptococcus pneumoniae* [32,33,34].

## 3. Nanoparticles in Adjuvanticity

As mentioned above, vaccination requires the delivery of vaccine antigens to the correct APCs in the correct intracellular compartment in order to induce specific adaptive immunity and the establishment of immunological memory. The use of particles in vaccine formulations could not only achieve improved antigen delivery (both by targeting APCs and by acting as an antigen depot), but it also has an important role in triggering immunity, *i.e.*, the adjuvant effect.

Adjuvanticity generally consists of a non-specific amplification of immune responses. In the case of induction of protective specific adaptive immunity to a vaccine antigen, the use of an adjuvant in the vaccine formulation induces a mild local inflammatory reaction that stimulates a much more efficient recruitment of immune cells and a quicker induction of adaptive immunity [2].

In the activity of particulated adjuvants, it is of particular interest that many of them apparently work by contributing to the activation of an intracytoplasmic protein complex, the inflammasome, which has the role of detecting stress signals and activating an enzyme involved in the triggering of inflammatory cytokines [35,36,37,38,39]. In particular, activation of the inflammasome causes the production of interkeukin-1β (IL-1β), an inflammatory cytokine that has a major immunostimulatory role [40,41,42].

**Figure 1:**
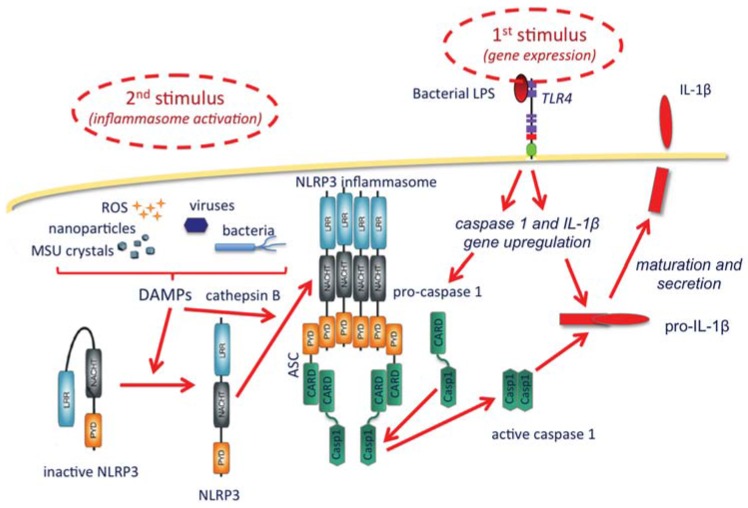
The NLRP3 activation and the production of IL-1β. A first stimulus, typically TLR agonists such as bacterial LPS (**upper right**), starts the activation by upregulating the expression of the genes encoding IL-1β and caspase-1, which are synthesized as inactive precursors (pro-caspase-1 and pro-IL-1β). The inflammasome is activated by a second type of stimuli (**upper left**), occurring intracellularly and being either endogenous or exogenous (particles and crystals of various origin, microorganisms, and reactive oxygen species), collectively dubbed danger-associated molecular patterns (DAMPs). These are able to change the inactive conformation of NLRP3, thus allowing the complexing of a number of NLRP3 molecules, an event facilitated by cathepsin B (released from ruptured lysosomes). The inflammasome assembles with the recruitment of adaptor proteins (in this case ASC), which in turn can take in the pro-caspase-1 and induce its cleavage and activation. Active caspase-1 then cleaves and helps the secretion of mature active IL-1β.

As shown in the Figure 1, the inflammasome is a protein complex with the role of activating caspase-1, the enzyme that matures and allows the extracellular release of IL-1β. IL-1β production broadly includes two steps, the first being its gene upregulation, which is typically triggered by microbial agents binding to membrane TLR receptors, such as the TLR4 agonist bacterial lipopolysaccharide (LPS). Upon gene upregulation, IL-1β is synthesized as a long, inactive pro-cytokine (pro-IL-1β), which must be cleaved by the enzyme caspase-1 to become active. Caspase-1 is also synthesized in an inactive form and needs cleavage to become active. The inflammasome complex, which recruits and activates caspase-1, includes a member of the NLRP family (Nucleotide-binding oligomerization domain, Leucine rich Repeat and Pyrin domain containing Proteins), the most abundant being NLRP3. NLRP3 is present in the cytoplasm in an inactive form that cannot complex into the inflammasome if not activated. Although the exact mechanisms are still being investigated, it is clear that activation of the inflammasome is brought about by a series of stimuli that include particles of different types, both exogenous and endogenous (bacteria, viruses, monosodium urate crystals, calcium pyrophosphate dihydrate crystals, alum, silica, asbestos, hyaluronan, protein aggregates, amyloid β, *etc.*). The effect of such particles is not direct and it is probably mediated by lysosomal rupture and release of cathepsin B, reactive oxygen species produced by stressed mitochondria, and K^+^ efflux [43]. A synthetic depiction of some of the known mechanisms of IL-1β production through inflammasome activation is reported in Figure 1. 

From the notions mentioned above, nanovaccination studies are taking a two-pronged approach. On the one hand, NPs can be modified with surface TLR ligands, so as to become able to efficiently bind and activate TLR receptors. A precursor of this concept is the adjuvant AS04, which is a TLR4 ligand (MPL) on alum particles. In addition, NPs can be shaped or modified in a way that facilitates phagolysosomal rupture once taken up by APCs. This is the case of crystalline particles (e.g., alum) or membrane-active particles (e.g., the protonic sponge particles). Thus, phagolysosomal rupture has the double scope of enhancing cross-presentation (consequent to the antigen escape to the cytoplasm) and of releasing inflammasome-activating molecules (e.g., cathepsin B). NPs with these characteristics (IL-1β gene upregulation, inflammasome activation) are therefore able to deliver to the cells all the stimuli required for the production of IL-1β, and they can act as very effective adjuvants. However, one should bear in mind that inflammasome activation and IL-1β production are also major players in a huge variety of diseases, including acute and chronic inflammatory diseases, degenerative diseases, autoimmune diseases, cancer, and many others [44]. For this reason, despite its excellent immunostimulatory activity, IL-1β has never become an immuno-enhancing drug or a vaccine adjuvant. The boundary between local beneficial (protective) inflammation and pathological inflammation is subtle, and is mostly based on the duration/persistence of the stimulus. Thus, when designing NPs endowed with adjuvant capacity, one needs to take into account the rate of degradation of the particles within the cells, which should be long enough to allow good antigen uptake, processing, and presentation, but not so long as to induce chronic inflammasome activation and pathological unresolved inflammation. Many studies are being conducted in the attempt to optimize NP-based adjuvants, but the safety issues of new inflammation-inducing particles still need a satisfactory solution [45,46].

## 4. Conclusions

The application of nanotechnology to vaccination exploits the capacity of NPs to function both as antigen carriers, able to deliver the vaccine antigen to the antigen-presenting cells, and as adjuvants, *i.e.*, amplifiers of immunity. The concept of using particles for improving immune response to vaccines and inducing effective immune protection is old, and it is based on the capacity of the immune system to recognize particulate agents with high efficiency. In comparison with the old-fashioned particle-based vaccines, nanotechnology offers the possibility of important advancements in the design of new vaccines, which may be summarized as follows:

1. Facilitating the targeting to the cells of the mononuclear phagocyte system, which are the major APCs, because of the NP particulate nature;
2. Possibility of precision targeting to the selected APCs by inserting specific molecules on the NP surface (ligands, receptors);
3. Possibility of directing intracellular localization to specific compartments in order to facilitate antigen presentation in the context of a selected MHC type or both types (cross-presentation);
4. Possibility of amplifying the establishment of adaptive protective immunity by inducing a localized and limited inflammatory reaction (controlled inflammasome activation and IL-1β production), an effect that could be increased by NP surface decoration with TLR ligands.

Current studies addressing the broad issues listed above are taking into particular consideration the fact that the NP design should be such as to obtain optimal efficacy in the absence of detrimental effects. This is an important issue as, for instance, the NP-based vaccines that work well in healthy adults may have less efficacy and more side effects when confronted with the “frail” immune reactivity of elderly people or people with chronic diseases [47]. Thus, the future perspective is that of precision vaccinology, *i.e.*, the design of personalized vaccination strategies that could obtain maximal efficacy and minimal collateral effects, based on the individual immune status. From this perspective, NP-based vaccines could play a major role due to the possibility of designing and manipulating the NP characteristics in a practically endless fashion.
